# Supplementary material for: The Systems Biology Research Tool: evolvable open-source software
Source: BMC Syst Biol. 2008 Jun 29;2:55. doi: 10.1186/1752-0509-2-55 (PMC2446383; doi:10.1186/1752-0509-2-55)
Supplement: Additional file 1 — SBRT Archive. An archive of the current version of the Systems Biology Research Tool. [file 1752-0509-2-55-S1.zip › sbrt-1.4.0/doc/developers_guide/api/sbrt/shell/io/package-frame.html]

sbrt.shell.io


sbrt.shell.io

Interfaces 
  
*InputFile*
  
*MapInputFile*
  
*MapOutputFile*
  
*MapTextFile*
  
*MapTextInputFile*
  
*MapTextOutputFile*
  
*OutputFile*
  
*SbrtFile*

Classes 
  
AbstractMapInputFile
  
AbstractMapOutputFile
  
AbstractSbrtFile
  
ComiFile
  
ComiFileSource
  
ComoFile
  
ComoFileSource
  
DevNull
  
EndOfFile
  
FileFormat
  
FileLineIterator
  
MultiOutputFiles
  
ProcessInputFile
  
RomiFile
  
RomiFileSource
  
RomoFile
  
RomoFileSource
  
SerializedFileIterator
  
SerializedInputFile
  
SerializedMapInputFile
  
SerializedOutputFile
  
SmoFile
  
SmoFileSource
  
TextFileLineIterator
  
TextInputFile
  
TextOutputFile
  
WorkingDirectory
  
XmlFileFactory
